# Reconceptualizing major depressive disorder as an infectious disease

**DOI:** 10.1186/2045-5380-4-10

**Published:** 2014-10-21

**Authors:** Turhan Canli

**Affiliations:** 1 Department of Psychology, Stony Brook University, Stony Brook, NY 11794, USA; 2 Department of Radiology, Stony Brook University, Stony Brook, NY 11794, USA; 3 Program in Genetics, Stony Brook University, Stony Brook, NY 11794, USA; 4 Program in Neuroscience, Stony Brook University, Stony Brook, NY 11794, USA

**Keywords:** Genomics, Virus, Bacterial, Parasite, Depression

## Abstract

In this article, I argue for a reconceptualization of major depressive disorder (major depression) as an infectious disease. I suggest that major depression may result from a parasitic, bacterial, or viral infection and present examples that illustrate possible pathways by which these microorganisms could contribute to the etiology of major depression. I also argue that the reconceptualization of the human body as an ecosystem for these microorganisms and the human genome as a host for non-human exogenous sequences may greatly amplify the opportunity to discover genetic links to the illness. Deliberately speculative, this article is intended to stimulate novel research approaches and expand the circle of researchers taking aim at this vexing illness.

## Background

Despite decades of substantial research efforts, major depressive disorder (MDD) remains among the most common mental disorders, with a 16.6% lifetime prevalence rate [1]. Pharmacological treatment approaches have not changed during this period, targeting primarily receptor-ligand interactions [2]. These types of antidepressants may bring relief to patients with severe symptoms but are not clinically more effective than placebos in mild to moderate cases [3]. Indeed, recurrence rates of 50% for first-episode patients and of 80% for second-episode patients [4] suggest that the core of the illness goes untreated.

Given this track record, I argue that it is time for an entirely different approach. Instead of conceptualizing MDD as an emotional disorder, I suggest to reconceptualize it as some form of an infectious disease. I propose that future research should conduct a concerted search for parasites, bacteria, or viruses that may play a causal role in the etiology of MDD. I present three arguments why this may be a fruitful endeavor. I have outlined the idea in much greater detail elsewhere [5], but will highlight some key points here.

### Main text

My first argument is that patients with MDD exhibit sickness behavior. Patients experience loss of energy; they commonly have difficulty getting out of bed and lose interest in the world around them. Although our Western conceptualization puts affective symptoms front-and-center, non-Western patients who meet DSM criteria for major depression report primarily somatic symptoms [6-11], reflecting in part cultural differences in the stigmatization of mental illness.

Yet, studies of inflammatory biomarkers in major depression strongly suggest an illness-related origin. For example, a meta-analysis of 24 studies confirmed prior reports of elevated TNFα and IL-6 in patients with major depression [12]. A second meta-analysis of 29 studies further extended the list of significantly elevated inflammatory markers to also include the soluble interleukin-2 receptor [13].

Several postmortem studies report the presence of inflammatory markers in the brains of depressed or mood-disordered patients. For example, compared to controls, female suicide victims showed elevated levels of IL-4 and male suicide victims showed elevated levels of IL-13 in Brodmann Area (BA) 11 [14], a brain region previously associated with suicidal ideation [15,16]. Compared to age-matched controls, patients diagnosed with major depression showed elevated levels of transmembrane TNFα (tmTNFα) in BA46 [17], a region associated with emotion regulation [18-20]. Patients with major depression, relative to controls, showed differential expression of a large set of both anti- and pro-inflammatory markers (including IL1α, 2, 3, 5, 8, 9, 10, 12A, 13, 15, 18, and IFNγ) in BA10 [21], a region associated with reward processing [22].

These inflammatory markers may represent activation of the immune system in response to some kind of pathogen, which could be a parasite, bacterium, or virus, and which could play a causal role in the etiology of depression. There is currently no direct evidence that major depression is caused by such microorganisms, but nature has offered some examples to illustrate that such a process is conceivable.

Thus, my second argument is that nature has already provided examples by which parasites, bacteria, or viruses can affect emotional behavior. The best-known example of a parasite that affects emotional behavior and that is relevant to human health is *Toxoplasma gondii. T. gondii* lives in the feline intestinal tract, where it lays its eggs, which are dispersed into the environment upon excretion. When a rat comes in contact with these eggs and becomes infected, it becomes attracted to the scent of cat urine [23,24]. This manipulation of the rat’s behavior involves the deposit of parasitic cysts across the rodent brain including the amygdala [25]. The mechanism for loss of fear to the scent of cat urine appears to involve a reduction in circulating corticosterone and dendritic retraction in the basolateral amygdala [26]. The mechanism for the rat’s attraction to the odor may involve activation of sexual arousal pathways [27].

The specificity of the behavioral change in the rat’s behavior appears to reflect functional changes that are limited to catecholaminergic neurons [28]. Infected animals have elevated levels of dopamine [29], but *T. gondii* can only synthesize tyrosine hydroxylase (which converts tyrosine to L-DOPA), and would therefore need to rely on catecholaminergic neurons to provide the needed DOPA decarboxylase to convert the L-DOPA to dopamine.

Human exposure to *T. gondii* is pervasive, with one-third of the world’s population [30] and one-fifth of the U.S. population [31] believed to be infected. Infection is associated with elevated inflammatory cytokines IL-6, IL-12, TNF, and IFN-γ [32,33], similarly as observed in depressed patients. A study of 20 European countries reported a positive correlation between *T. gondii* prevalence rates and national suicide rates [34]. Among patients with diagnosed major depression or bipolar disorder, those with a history of suicide attempt had higher *T. gondii* antibody titers [35]. Yet, large-scale studies of major depression and *T. gondii* or systematic searches to discover other potential parasitic infections have not yet been conducted.

Bacteria could be another causal factor for major depression. Studies of bacterial colonies residing in the gastrointestinal tract have begun to examine links to emotional behavior. In the first study of this kind, germ-free (GF), specific pathogen-free (SPF), and gnotobiotic mice were compared in their response to restraint stress [36]. GF mice exhibited higher levels of plasma ACTH and corticosterone and had lower levels of brain-derived neurotrophic factor in the cortex and hippocampus, compared to SPF mice. The elevated stress response of GF mice was normalized with administration of the bacterium *Bifidobacterium infantis*. Another rodent study showed that administration of *B. infantis* in rats reduced the levels of IFN-γ, TNF-α, and IL-6 following mitogen stimulation and altered tryptophan, 5-HIAA, and DOPAC levels in the frontal cortex and amygdala [37]. Administration of the *Lactobacillus rhamnosus* strain in mice was shown to alter GABAergic expression in the brain: elevating GABA_B1b_ mRNA in the cingulate and prelimbic cortices, while reducing it in hippocampus and amygdala, among other regions [38].

The ‘leaky gut’ hypothesis proposes a mechanism by which gastrointestinal bacteria may contribute to major depression [39,40]. According to this hypothesis, cytokines or other stressors may render the intestinal tract permeable to lipopolysaccharides (LPS) from gram-negative bacteria to activate the immune system. Indeed, the model is supported by data showing elevated serum concentrations of IgM and IgA against LPS of the gram-negative enterobacteria in depressed patients [39,40]. These studies were conducted with relatively small numbers of patients and suggested that this mechanism may apply to some subgroups of patients but not others. It would be useful to expand the search using large patient cohorts and a wide range of different antibodies. Future work should then examine potential neural mechanisms.

Viruses represent the third pathogenic route in the etiology of major depression. A meta-analysis of 28 studies explicitly examined the link between infectious agents and depression [41]. Among viruses that had significant associations with the illness were the Borna disease virus (BDV), herpes simplex virus-1, varicella zoster virus, and Epstein-Barr virus. Among these, BDV has been studied most extensively and was 3.25 times more likely to be found in depressed patients than in normal controls [41]. One postmortem study reported BDV infection in 2 out of 30 depressed patients in the frontal and temporal cortex, olfactory bulb, and hippocampus [42], although a larger study failed to detect any infection [43]. A small open-label study of BDV-infected depressed patients reported a reduction in both depressive symptoms and BDV infection upon treatment with the antiviral drug amantadine [44].

The mechanism between BDV infection and depression could involve glutaminergic transmission, because amantadine is an antagonist of the N-methyl-D-aspartate (NMDA) receptor, one of the receptors targeted by glutamate. The related NMDA antagonist memantine has been evaluated in a randomized, double-blind study of patients diagnosed with bipolar depression, where it was applied to augment treatment with the presynaptic glutamate release inhibitor lamotrigine, and found to accelerate treatment response [45]. Another NMDA receptor antagonist, ketamine, also has antidepressant effects [46], which appear to be mediated by changes in mTOR signaling [47]. However, the literature on BDV infection and depression remains controversial, with several studies failing to replicate any association between the two [48-51].

My third argument is that reconceptualizing major depression as being causally linked to parasites, bacteria, or viruses is useful when thinking about the genetics of this illness. Evidence from twin studies notwithstanding, the search for specific genes linked to major depression has come up empty [52,53]. Perhaps, we have been looking at the wrong organism. Genetic studies to date have focused the search on human genes within our genome. Yet, 8% of the human genome is based on exogenous sequences from retroviruses [54]. These retroviral insertions may sometimes benefit the human host and therefore be protected from mutational degeneration [55]. Indeed, the BDV discussed earlier inserted some of its sequences into vertebrate genomes approximately 40 million years ago [56], and presence of these sequences correlates with disease resistance to BDV. Parasites could also add exogenous sequences to the human genome through the process of horizontal gene transfer [57]. It is possible that polymorphisms within such exogenous sequences, or interactions between these exogenous sequences and other variables such as human gene polymorphisms or stressful life experiences, could render some individuals vulnerable to major depression.

Furthermore, if we view the human body as an ecosystem that is a host to numerous microorganisms which may be passed across generations, the opportunity for genetic discoveries is vastly amplified. For example, an estimated thousand species of bacteria reside in the human gastrointestinal tract [58], and these could be passed during birth or through common environmental exposure between parents and offspring [59]. Humans also carry vast numbers of viruses, which can be unknown and go undetected until subjected to a concerted search using new approaches such as deep sequencing [60].

## Conclusions

In light of the above considerations, an important point of reflection concerns the relation between the immune response and MDD and the specificity of any putative mechanism. The literature implicating the immune system in MDD [61] can be read as suggesting that the immune response itself is the causal mechanism in depression. Indeed, conditions such as hypoxia known to produce sterile inflammation ([62], i.e., activation of the immune system sans a pathogen) may increase the risk of depression [61] in conditions such as obstructive sleep apnea [63] or chronic obstructive pulmonary disease [64]. Yet, most cases of MDD are not attributable to sterile inflammation. Thus, I suggest that some unknown pathogen(s) could play a causal role, and that the immune response is secondary to the infection; interventions that only target the immune response may bring symptom relief but would not address the root cause of the illness.

If a pathogen played a causal role in MDD, the next question would concern the specificity of the mechanism. One perspective would favor a very general, non-specific mechanism. For example, chronic fatigue syndrome (CFS)—which is characterized by sickness behavior that may include depressive symptoms—has been hypothesized to be caused by vagus nerve infection, regardless of the type of pathogen [65]. My view is that, for MDD, the type of pathogen may matter a great deal, and that it plays a very specific causal role: the examples I presented above suggest plausible mechanisms by which pathogens may alter neurotransmission. However, there may not be a single pathogen that causes all cases of MDD. Instead, there may be a class of pathogens, similar to those discussed above, which share common modes of action. This class of pathogens would specifically target the nervous system in a manner that causally contributes to MDD. I use the term ‘contribute’ to indicate that these pathogens may act in concert with other variables. For example, an individual may carry a latent infection and be asymptomatic for depressive symptoms. This individual would be characterized by susceptibility to MDD which may only emerge after the pathogen was activated by other factors such as stressful life events; this activation could then also trigger a concomitant immune response. It is possible that such a pathogen-driven mechanism is not limited to MDD but may contribute to other psychopathologies. For example, posttraumatic stress disorder could be one such extension of the same mechanism: not every individual develops the disorder in response to a traumatic experience (suggesting individual differences in susceptibility), and the illness is accompanied by immune system activation [66,67].

In closing, I think it would be worthwhile to conduct large-scale studies of carefully characterized depressed patients and healthy controls, using gold-standard clinical and infectious disease-related study protocols, as have already been developed for bacteria [68,69] and viruses [70-76]. Such efforts, if successful, would represent the ‘end of the beginning’ , as any such discovery would represent the first step toward developing a vaccination for major depression.

## Abbreviations

BA: Brodmann Area; BDV: Borna disease virus; GABA: gamma-aminobutyric acid; IFN-γ: interferon gamma; IgA: immunoglobulin A; IgM: immunoglobulin M; IL: interleukin; L-DOPA: L -3,4-dihydroxyphenylalanine; LPS: lipopolysaccharides; MDD: major depressive disorder; NMDA: N-methyl-D-aspartate; TNFα: tumor necrosis factor alpha; tmTNFα: transmembrane tumor necrosis factor alpha.

## Competing interests

The author declares that he has no competing interests.

## References

[B1] Kessler RC Petukhova M Sampson NA Zaslavsky AM Wittchen H-U Twelve-month and lifetime prevalence and lifetime morbid risk of anxiety and mood disorders in the United States Int J Methods Psychiatr Res 2012 21 169 184 10.1002/mpr.1359 22865617 PMC4005415

[B2] Agid Y Buzsaki G Diamond DM Frackowiak R Giedd J Girault JA Grace A Lambert JJ Manji H Mayberg H Popoli M Prochiantz A Richter-Levin G Somogyi P Spedding M Svenningsson P Weinberger D How can drug discovery for psychiatric disorders be improved? Nat Rev Drug Discov 2007 6 189 201 10.1038/nrd2217 17330070

[B3] Kirsch I Deacon BJ Huedo-Medina TB Scoboria A Moore TJ Johnson BT Initial severity and antidepressant benefits: a meta-analysis of data submitted to the food and drug administration PLoS Med 2008 5 e45 10.1371/journal.pmed.0050045 18303940 PMC2253608

[B4] Burcusa SL Iacono WG Risk for recurrence in depression Clin Psychol Rev 2007 27 959 985 10.1016/j.cpr.2007.02.005 17448579 PMC2169519

[B5] Canli T Canli T Is depression an infectious disease? The Oxford Handbook of Molecular Psychology 2014 New York: Oxford University Press Online Publication Date: Sep 2014. doi:10.1093/oxfordhb/9780199753888.013.28. [http://www.oxfordhandbooks.com/view/10.1093/oxfordhb/9780199753888.001.0001/oxfordhb-9780199753888-e-28]

[B6] Seifsafari S Firoozabadi A Ghanizadeh A Salehi A A symptom profile analysis of depression in a sample of Iranian patients Iran J Med Sci 2013 38 22 29 23645954 PMC3642941

[B7] Wilkowska-Chmielewska J Szelenberger W Wojnar M Age-dependent symptomatology of depression in hospitalized patients and its implications for DSM-5 J Affect Disord 2013 150 142 145 10.1016/j.jad.2012.12.012 23332650

[B8] Raguram R Weiss MG Channabasavanna SM Devins GM Stigma, depression, and somatization in South India Am J Psychiatry 1996 153 1043 1049 8678173 10.1176/ajp.153.8.1043

[B9] Rao D Young M Raguram R Culture, somatization, and psychological distress: symptom presentation in South Indian patients from a public psychiatric hospital Psychopathology 2007 40 349 355 10.1159/000106312 17657134

[B10] Heredia Montesinos A Rapp MA Temur-Erman S Heinz A Hegerl U Schouler-Ocak M The influence of stigma on depression, overall psychological distress, and somatization among female Turkish migrants Eur Psychiatry 2012 27 Suppl 2 S22 S26 22863246 10.1016/S0924-9338(12)75704-8

[B11] Bagayogo IP Interian A Escobar JI Transcultural aspects of somatic symptoms in the context of depressive disorders Adv Psychosom Med 2013 33 64 74 23816864 10.1159/000350057

[B12] Dowlati Y Herrmann N Swardfager W Liu H Sham L Reim EK Lanctot KL A meta-analysis of cytokines in major depression Biol Psychiatry 2010 67 446 457 10.1016/j.biopsych.2009.09.033 20015486

[B13] Liu Y Ho RC Mak A Interleukin (IL)-6, tumour necrosis factor alpha (TNF-alpha) and soluble interleukin-2 receptors (sIL-2R) are elevated in patients with major depressive disorder: a meta-analysis and meta-regression J Affect Disord 2012 139 230 239 10.1016/j.jad.2011.08.003 21872339

[B14] Tonelli LH Stiller J Rujescu D Giegling I Schneider B Maurer K Schnabel A Moller HJ Chen HH Postolache TT Elevated cytokine expression in the orbitofrontal cortex of victims of suicide Acta Psychiatr Scand 2008 117 198 206 10.1111/j.1600-0447.2007.01128.x 18081924 PMC2612100

[B15] Mann JJ Neurobiology of suicidal behaviour Nat Rev Neurosci 2003 4 819 828 14523381 10.1038/nrn1220

[B16] Oquendo MA Placidi GP Malone KM Campbell C Keilp J Brodsky B Kegeles LS Cooper TB Parsey RV van Heertum RL Mann JJ Positron emission tomography of regional brain metabolic responses to a serotonergic challenge and lethality of suicide attempts in major depression Arch Gen Psychiatry 2003 60 14 22 10.1001/archpsyc.60.1.14 12511168

[B17] Dean B Tawadros N Scarr E Gibbons AS Regionally-specific changes in levels of tumour necrosis factor in the dorsolateral prefrontal cortex obtained postmortem from subjects with major depressive disorder J Affect Disord 2010 120 245 248 10.1016/j.jad.2009.04.027 19446343

[B18] Silvers JA Weber J Wager TD Ochsner KN Bad and worse: neural systems underlying reappraisal of high- and low-intensity negative emotions Soc Cogn Affect Neuroscifirst published online March 5, 2014 doi:10.1093/scan/nsu043 10.1093/scan/nsu043 PMC4321618 24603024

[B19] Belden AC Luby JL Pagliaccio D Barch DM Neural activation associated with the cognitive emotion regulation of sadness in healthy children Dev Cogn Neurosci 2014 9 136 147 24646887 10.1016/j.dcn.2014.02.003 PMC4061244

[B20] Kohn N Eickhoff SB Scheller M Laird AR Fox PT Habel U Neural network of cognitive emotion regulation–an ALE meta-analysis and MACM analysis Neuroimage 2014 87 345 355 24220041 10.1016/j.neuroimage.2013.11.001 PMC4801480

[B21] Shelton RC Claiborne J Sidoryk-Wegrzynowicz M Reddy R Aschner M Lewis DA Mirnics K Altered expression of genes involved in inflammation and apoptosis in frontal cortex in major depression Mol Psychiatry 2011 16 751 762 10.1038/mp.2010.52 20479761 PMC2928407

[B22] Rogers RD Owen AM Middleton HC Williams EJ Pickard JD Sahakian BJ Robbins TW Choosing between small, likely rewards and large, unlikely rewards activates inferior and orbital prefrontal cortex J Neurosci 1999 19 9029 9038 10516320 10.1523/JNEUROSCI.19-20-09029.1999 PMC6782753

[B23] Berdoy M Webster JP Macdonald DW Fatal attraction in rats infected with Toxoplasma gondii Proc Biol Sci Royal Soc 2000 267 1591 1594 10.1098/rspb.2000.1182 PMC1690701 11007336

[B24] Vyas A Kim SK Giacomini N Boothroyd JC Sapolsky RM Behavioral changes induced by Toxoplasma infection of rodents are highly specific to aversion of cat odors Proc Natl Acad Sci U S A 2007 104 6442 6447 10.1073/pnas.0608310104 17404235 PMC1851063

[B25] Berenreiterova M Flegr J Kubena AA Nemec P The distribution of Toxoplasma gondii cysts in the brain of a mouse with latent toxoplasmosis: implications for the behavioral manipulation hypothesis PLoS One 2011 6 e28925 10.1371/journal.pone.0028925 22194951 PMC3237564

[B26] Mitra R Sapolsky RM Vyas A Toxoplasma gondii infection induces dendritic retraction in basolateral amygdala accompanied by reduced corticosterone secretion Dis Model Mech 2013 6 516 520 10.1242/dmm.009928 23104989 PMC3597033

[B27] House PK Vyas A Sapolsky R Predator cat odors activate sexual arousal pathways in brains of Toxoplasma gondii infected rats PLoS One 2011 6 e23277 10.1371/journal.pone.0023277 21858053 PMC3157360

[B28] McConkey GA Martin HL Bristow GC Webster JP Toxoplasma gondii infection and behaviour - location, location, location? J Exp Biol 2013 216 113 119 10.1242/jeb.074153 23225873 PMC3515035

[B29] Stibbs HH Changes in brain concentrations of catecholamines and indoleamines in Toxoplasma gondii infected mice Ann Trop Med Parasitol 1985 79 153 157 2420295 10.1080/00034983.1985.11811902

[B30] Montoya JG Liesenfeld O Toxoplasmosis Lancet 2004 363 1965 1976 10.1016/S0140-6736(04)16412-X 15194258

[B31] Centers for Disease Control and Prevention (CDC) Parasites - toxoplasmosis (toxoplasma infection) [http://www.cdc.gov/parasites/toxoplasmosis/epi.html]

[B32] Hsu PC Groer M Beckie T New findings: depression, suicide, and toxoplasma gondii infection J Am Assoc Nurse Prac 2014 [http://dx.doi.org/10.1002/2327-6924.12129] 10.1002/2327-6924.12129 24715687

[B33] Munoz M Liesenfeld O Heimesaat MM Immunology of toxoplasma gondii Immunol Rev 2011 240 269 285 10.1111/j.1600-065X.2010.00992.x 21349099

[B34] Lester D Brain parasites and suicide Psychol Rep 2010 107 424 10.2466/12.13.PR0.107.5.424 21117467

[B35] Arling TA Yolken RH Lapidus M Langenberg P Dickerson FB Zimmerman SA Balis T Cabassa JA Scrandis DA Tonelli LH Postolache TT Toxoplasma gondii antibody titers and history of suicide attempts in patients with recurrent mood disorders J Nerv Ment Dis 2009 197 905 908 10.1097/NMD.0b013e3181c29a23 20010026

[B36] Sudo N Chida Y Aiba Y Sonoda J Oyama N Yu XN Kubo C Koga Y Postnatal microbial colonization programs the hypothalamic-pituitary-adrenal system for stress response in mice J Physiol 2004 558 263 275 10.1113/jphysiol.2004.063388 15133062 PMC1664925

[B37] Desbonnet L Garrett L Clarke G Bienenstock J Dinan TG The probiotic Bifidobacteria infantis: an assessment of potential antidepressant properties in the rat J Psychiatr Res 2008 43 164 174 10.1016/j.jpsychires.2008.03.009 18456279

[B38] Bravo JA Forsythe P Chew MV Escaravage E Savignac HM Dinan TG Bienenstock J Cryan JF Ingestion of lactobacillus strain regulates emotional behavior and central GABA receptor expression in a mouse via the vagus nerve Proc Natl Acad Sci U S A 2011 108 16050 16055 10.1073/pnas.1102999108 21876150 PMC3179073

[B39] Maes M Kubera M Leunis JC Berk M Increased IgA and IgM responses against gut commensals in chronic depression: further evidence for increased bacterial translocation or leaky gut J Affect Disord 2012 141 55 62 10.1016/j.jad.2012.02.023 22410503

[B40] Maes M Kubera M Leunis JC The gut-brain barrier in major depression: intestinal mucosal dysfunction with an increased translocation of LPS from gram negative enterobacteria (leaky gut) plays a role in the inflammatory pathophysiology of depression Neuro endocrinol Letters 2008 29 117 124 18283240

[B41] Wang X Zhang L Lei Y Liu X Zhou X Liu Y Wang M Yang L Zhang L Fan S Xie P Meta-analysis of infectious agents and depression Sci Reports 2014 4 4530 10.1038/srep04530 PMC5380112 24681753

[B42] Haga S Yoshimura M Motoi Y Arima K Aizawa T Ikuta K Tashiro M Ikeda K Detection of Borna disease virus genome in normal human brain tissue Brain Res 1997 770 307 309 10.1016/S0006-8993(97)00903-7 9372235

[B43] Czygan M Hallensleben W Hofer M Pollak S Sauder C Bilzer T Blumcke I Riederer P Bogerts B Falkai P Schwarz MJ Masliah E Staeheli P Hufert FT Lieb K Borna disease virus in human brains with a rare form of hippocampal degeneration but not in brains of patients with common neuropsychiatric disorders J Infect Dis 1999 180 1695 1699 10.1086/315068 10515835

[B44] Dietrich DE Bode L Human Borna disease virus-infection and its therapy in affective disorders APMIS Suppl 2008 124 61 65 18771101 10.1111/j.1600-0463.2008.00m10.x

[B45] Stevens J Bies RR Shekhar A Anand A Bayesian model of Hamilton depression rating score (HDRS) with memantine augmentation in bipolar depression Br J Clin Pharmacol 2013 75 791 798 22846159 10.1111/j.1365-2125.2012.04398.x PMC3575945

[B46] Berman RM Cappiello A Anand A Oren DA Heninger GR Charney DS Krystal JH Antidepressant effects of ketamine in depressed patients Biol Psychiatry 2000 47 351 354 10.1016/S0006-3223(99)00230-9 10686270

[B47] Li N Lee B Liu RJ Banasr M Dwyer JM Iwata M Li XY Aghajanian G Duman RS mTOR-dependent synapse formation underlies the rapid antidepressant effects of NMDA antagonists Science 2010 329 959 964 10.1126/science.1190287 20724638 PMC3116441

[B48] Na KS Tae SH Song JW Kim YK Failure to detect Borna disease virus antibody and RNA from peripheral blood mononuclear cells of psychiatric patients Psychiatry Investig 2009 6 306 312 10.4306/pi.2009.6.4.306 20140130 PMC2808801

[B49] Hornig M Briese T Licinio J Khabbaz RF Altshuler LL Potkin SG Schwemmle M Siemetzki U Mintz J Honkavuori K Kraemer HC Egan MF Whybrow PC Bunney WE Lipkin WI Absence of evidence for bornavirus infection in schizophrenia, bipolar disorder and major depressive disorder Mol Psychiatry 2012 17 486 493 10.1038/mp.2011.179 22290118 PMC3622588

[B50] Iwata Y Takahashi K Peng X Fukuda K Ohno K Ogawa T Gonda K Mori N Niwa S Shigeta S Detection and sequence analysis of borna disease virus p24 RNA from peripheral blood mononuclear cells of patients with mood disorders or schizophrenia and of blood donors J Virol 1998 72 10044 10049 9811743 10.1128/jvi.72.12.10044-10049.1998 PMC110530

[B51] Kim YK Kim SH Choi SH Ko YH Kim L Lee MS Suh KY Kwak DI Song KJ Lee YJ Yanagihara R Song JW Failure to demonstrate Borna disease virus genome in peripheral blood mononuclear cells from psychiatric patients in Korea J Neurovirol 1999 5 196 199 10.3109/13550289909022002 10321984

[B52] Lewis CM Ng MY Butler AW Cohen-Woods S Uher R Pirlo K Weale ME Schosser A Paredes UM Rivera M Craddock N Owen MJ Jones L Jones I Korszun A Aitchison KJ Shi J Quinn JP Mackenzie A Vollenweider P Waeber G Heath S Lathrop M Muglia P Barnes MR Whittaker JC Tozzi F Holsboer F Preisig M Farmer AE Genome-wide association study of major recurrent depression in the U.K. population Am J Psychiatry 2010 167 949 957 10.1176/appi.ajp.2010.09091380 20516156

[B53] Verbeek EC Bakker IM Bevova MR Bochdanovits Z Rizzu P Sondervan D Willemsen G de Geus EJ Smit JH Penninx BW Boomsma DI Hoogendijk WJ Heutink P A fine-mapping study of 7 top scoring genes from a GWAS for major depressive disorder PLoS One 2012 7 e37384 10.1371/journal.pone.0037384 22649524 PMC3359349

[B54] Lander ES Linton LM Birren B Nusbaum C Zody MC Baldwin J Devon K Dewar K Doyle M FitzHugh W Funke R Gage D Harris K Heaford A Howland J Kann L Lehoczky J LeVine R McEwan P McKernan K Meldrim J Mesirov JP Miranda C Morris W Naylor J Raymond C Rosetti M Santos R Sheridan A Sougnez C Initial sequencing and analysis of the human genome Nature 2001 409 860 921 10.1038/35057062 11237011

[B55] Kurdyukov SG Lebedev YB Artamonova II Gorodentseva TN Batrak AV Mamedov IZ Azhikina TL Legchilina SP Efimenko IG Gardiner K Sverdlov ED Full-sized HERV-K (HML-2) human endogenous retroviral LTR sequences on human chromosome 21: map locations and evolutionary history Gene 2001 273 51 61 10.1016/S0378-1119(01)00570-4 11483360

[B56] Belyi VA Levine AJ Skalka AM Unexpected inheritance: multiple integrations of ancient bornavirus and ebolavirus/marburgvirus sequences in vertebrate genomes PLoS Pathog 2010 6 e1001030 10.1371/journal.ppat.1001030 20686665 PMC2912400

[B57] Gilbert C Schaack S Pace JK 2nd Brindley PJ Feschotte C A role for host-parasite interactions in the horizontal transfer of transposons across phyla Nature 2010 464 1347 1350 10.1038/nature08939 20428170 PMC3004126

[B58] Cryan JF Dinan TG Mind-altering microorganisms: the impact of the gut microbiota on brain and behaviour Nat Rev Neurosci 2012 13 701 712 10.1038/nrn3346 22968153

[B59] Yatsunenko T Rey FE Manary MJ Trehan I Dominguez-Bello MG Contreras M Magris M Hidalgo G Baldassano RN Anokhin AP Heath AC Warner B Reeder J Kuczynski J Caporaso JG Lozupone CA Lauber C Clemente JC Knights D Knight R Gordon JI Human gut microbiome viewed across age and geography Nature 2012 486 222 227 22699611 10.1038/nature11053 PMC3376388

[B60] Wylie KM Mihindukulasuriya KA Sodergren E Weinstock GM Storch GA Sequence analysis of the human virome in febrile and afebrile children PLoS One 2012 7 e27735 10.1371/journal.pone.0027735 22719819 PMC3374612

[B61] Dantzer R O’Connor JC Freund GG Johnson RW Kelley KW From inflammation to sickness and depression: when the immune system subjugates the brain Nat Rev Neurosci 2008 9 46 56 10.1038/nrn2297 18073775 PMC2919277

[B62] Johnson DR O’Connor JC Hartman ME Tapping RI Freund GG Acute hypoxia activates the neuroimmune system, which diabetes exacerbates J Neurosci 2007 27 1161 1166 10.1523/JNEUROSCI.4560-06.2007 17267571 PMC6673177

[B63] Ohayon MM The effects of breathing-related sleep disorders on mood disturbances in the general population J Clin Psychiatry 2003 64 1195 1200 quiz, 1274–1196 10.4088/JCP.v64n1009 14658968

[B64] Regvat J Zmitek A Vegnuti M Kosnik M Suskovic S Anxiety and depression during hospital treatment of exacerbation of chronic obstructive pulmonary disease J Int Med Res 2011 39 1028 1038 10.1177/147323001103900338 21819737

[B65] VanElzakker MB Chronic fatigue syndrome from vagus nerve infection: a psychoneuroimmunological hypothesis Med Hypotheses 2013 81 414 423 10.1016/j.mehy.2013.05.034 23790471

[B66] Hoge EA Brandstetter K Moshier S Pollack MH Wong KK Simon NM Broad spectrum of cytokine abnormalities in panic disorder and posttraumatic stress disorder Depress Anxiety 2009 26 447 455 10.1002/da.20564 19319993

[B67] Pace TWW Heim CM A short review on the psychoneuroimmunology of posttraumatic stress disorder: from risk factors to medical comorbidities Brain Behav Immun 2011 25 6 13 10.1016/j.bbi.2010.10.003 20934505

[B68] Kuczynski J Lauber CL Walters WA Parfrey LW Clemente JC Gevers D Knight R Experimental and analytical tools for studying the human microbiome Nat Rev Genet 2012 13 47 58 22179717 10.1038/nrg3129 PMC5119550

[B69] Human Microbiome Project C A framework for human microbiome research Nature 2012 486 215 221 10.1038/nature11209 22699610 PMC3377744

[B70] Barber RM Porter BF Li Q May M Claiborne MK Allison AB Howerth EW Butler A Wei S Levine JM Levine GJ Brown DR Schatzberg SJ Broadly reactive polymerase chain reaction for pathogen detection in canine granulomatous meningoencephalomyelitis and necrotizing meningoencephalitis J Vet Intern Med 2012 26 962 968 10.1111/j.1939-1676.2012.00954.x 22686439 PMC7166683

[B71] Allen LZ Ishoey T Novotny MA McLean JS Lasken RS Williamson SJ Single virus genomics: a new tool for virus discovery PLoS One 2011 6 e17722 10.1371/journal.pone.0017722 21436882 PMC3059205

[B72] Zeigler Allen L Ishoey T Novotny MA McLean JS Lasken RS Williamson SJ Isolation and genome analysis of single virions using ‘single virus genomics’ J Vis Exp 2013 75 e3899 doi:10.3791/3899 23728084 10.3791/3899 PMC3719269

[B73] Cook S Chung BY Bass D Moureau G Tang S McAlister E Culverwell CL Glucksman E Wang H Brown TD Gould EA Harbach RE de Lamballerie X Firth AE Novel virus discovery and genome reconstruction from field RNA samples reveals highly divergent viruses in dipteran hosts PLoS One 2013 8 e80720 10.1371/journal.pone.0080720 24260463 PMC3832450

[B74] Tang P Chiu C Metagenomics for the discovery of novel human viruses Future Microbiol 2010 5 177 189 10.2217/fmb.09.120 20143943

[B75] Kreuze JF Perez A Untiveros M Quispe D Fuentes S Barker I Simon R Complete viral genome sequence and discovery of novel viruses by deep sequencing of small RNAs: a generic method for diagnosis, discovery and sequencing of viruses Virology 2009 388 1 7 10.1016/j.virol.2009.03.024 19394993

[B76] Barzon L Lavezzo E Costanzi G Franchin E Toppo S Palu G Next-generation sequencing technologies in diagnostic virology J Clin Virol 2013 58 346 350 10.1016/j.jcv.2013.03.003 23523339

